# Gestational age–dependent risk patterns in small for gestational age preterm infants: a large multivariable cohort study

**DOI:** 10.3389/fped.2026.1877628

**Published:** 2026-08-27

**Authors:** Josep Figueras-Aloy, Xavier Carbonell-Estrany, Francesc Botet-Mussons, Óscar García-Algar, Martí Iriondo-Sanz, Giorgia Sebastiani

**Affiliations:** Neonatology Department, Center for Maternal-Fetal and Neonatal Medicine (BCNatal), Hospital Clínic and Hospital Sant Joan de Déu, University of Barcelona, Barcelona, Spain

**Keywords:** fetal growth restriction, neonatal morbidity, neonatal mortality, prematurity, small for gestational age

## Abstract

**Background:**

Small for gestational age (SGA) preterm infants remain at high risk for morbidity and mortality, yet the influence of gestational age (GA) on these outcomes is not fully understood.

**Methods:**

The influence of gestational age on morbidity and mortality in SGA preterm infants (23–34 weeks) was analyzed in a cohort of 11,208 preterm infants. Infants were divided into three groups according to gestational age (23–27 weeks, 28–31 weeks, and 32–34 weeks).

**Results:**

Compared with non-SGA infants of the same gestational age group, SGA infants showed higher rates of fetal distress and Apgar score ≤5 at any gestational age. Hyaline membrane disease increased between 23 and 27 weeks but decreased between 32 and 34 weeks, whereas intraventricular hemorrhage showed the opposite trend. Pneumothorax and periventricular leukomalacia increased mainly between 23 and 27 weeks, while necrotizing enterocolitis, sepsis, and gastrointestinal perforation increased between 28 and 31 weeks. Mortality was higher between 23 and 31 weeks. In survivors, moderate-severe bronchopulmonary dysplasia increased between 23 and 31 weeks, and retinopathy of prematurity increased after 28 weeks. Gestational age appeared to modify the associations between SGA status and selected neonatal outcomes, including hyaline membrane disease, intraventricular hemorrhage, and mortality.

**Conclusions:**

Gestational age may influence the pattern of associated morbidities among SGA preterm infants born at 23 to 34 weeks of gestation. In these newborns, the risk of mortality was increased 3.13-fold.

## Introduction

Small for gestational age (SGA) and fetal growth restriction (FGR) should not be considered synonymous. SGA is an anthropometric definition based on birth weight below a specified cutoff point, whereas FGR refers to a pathological process of impaired fetal growth. Although many SGA infants have FGR, others are constitutionally small, and some fetuses with FGR are born with birth weights above the cutoff point. In this study, SGA is defined as a newborn with birth weight below the 10th percentile for gestational age and sex, as determined by applying any of the neonatal growth charts (1, 2). The prevalence of SGA in a population sample depends on the neonatal growth charts used. These charts should include data from week 23 of gestation, as is the case with the 2013 Fenton chart (Fenton-13) (3), the 2021 Intergrowth chart (Intergrowth-21) (4) and the 2025 Fenton chart (Fenton-25) (5). We have used the Fenton-13 charts.

When SGA is associated with preterm birth at 34 weeks of gestation or less, the complications related to immaturity must also be considered. These infants have an increased risk of morbidity and mortality (6–8) as well as in neurological sequelae up to 3 years of age (9). However, it remains unclear which morbidities are most influenced by different gestational ages. A review of the literature frequently reveals discrepancies in the most common morbidities in SGA compared to non-SGA infants. The literature reports that, among preterm SGA infants, the risk of intraventricular hemorrhage is higher (10), similar (11), or even lower (12) than that observed in preterm non-SGA infants. Our hypothesis is that the diverse published findings are due to the different gestational ages of the SGA infants studied, since a given pathology could increase at one gestational age and decrease at a later one, or vice versa, showing a clear trend in this direction.

The aim of this study is to examine the influence of gestational age on morbidity and mortality related to SGA status in a large sample of preterm infants born between 23 and 34 weeks of gestation.

## Materials and methods

All preterm infants born between 23 and 34 weeks of gestation and treated between 1995 and 2023 at three Level III hospitals that follow the same obstetric and neonatal care protocols were included. These infants were from singleton or multiple pregnancies and were admitted alive to the neonatal unit or transferred from other centers. Neonates with major malformations, chromosomal abnormalities, and metabolic disorders were excluded. The project was approved by the clinical research ethics committee of one of the hospitals.

The study consisted of a review of the neonatal database, common to the three centers since 1995. The sample was divided into three groups according to gestational age: 23–27 weeks (extremely premature), 28–31 weeks (very premature) and 32–34 weeks (moderately premature). The dependent (study) variable was SGA status, defined as a birth weight below the 10th percentile for gestational age and sex, according to the Fenton-13 growth charts. For each included neonate, variables related to pregnancy and delivery were collected (single or multiple pregnancy, administration of prenatal corticosteroids, presence of preeclampsia, maternal diabetes or fetal distress and birth by cesarean section), the newborn (sex, Apgar score at one minute ≤5, need for vigorous resuscitation (with endotracheal intubation), birth weight and gestational age (determined by the obstetricians and recorded in weeks and days) and the pathology presented during the neonatal period: hyaline membrane disease (oxygen requirement >30% and compatible findings on chest radiography or lung ultrasound), pneumothorax (typical findings on chest radiography or lung ultrasound), intraventricular hemorrhage (any grade), periventricular leukomalacia (cystic or echogenic), patent ductus arteriosus (clinically significant with echocardiographic confirmation), sepsis (early- or late-onset) with positive blood culture, acute renal failure (urine output <1 mL/kg/hour for 12 h or serum creatinine >1.2 mg/dL), necrotizing enterocolitis (Bell stage ≥2), gastrointestinal perforation (pneumoperitoneum with surgical confirmation), and death during hospital admission. Among survivors, the need of supplemental oxygen or non-invasive ventilation (NIV) at 36 weeks postmenstrual age (moderate-severe bronchopulmonary dysplasia) and the presence of any stage of retinopathy of prematurity were recorded.

Quantitative variables did not follow a normal distribution (Kolmogorov–Smirnov test), and non-parametric tests were used. Descriptive statistics for quantitative variables are presented as medians and interquartile ranges (25th to 75th percentiles). Qualitative variables are expressed as percentages. Quantitative variables were compared using the Mann–Whitney U test or the Kruskal–Wallis H test. Incidences (qualitative variables) were compared using the chi-square test or Fisher's exact test, as appropriate, and the odds ratio (OR) for the variable present in cases of SGA was also determined, with a 95% confidence interval. Logistic regression models including an interaction term between birth weight status (SGA vs. non-SGA) and gestational age group (three groups) were performed using the enter method and adjusted for potential confounders. Interaction *p*-values were reported for all morbidity outcomes and mortality. For outcomes with statistically significant interaction, measures of additive interaction, including the relative excess risk due to interaction (RERI), attributable proportion due to interaction (AP), and synergy index that compares the observed combined effect with the expected effect under an additive model (S) and their 95% confidence intervals were estimated using the delta method. Logistic regression was used as a multivariate analysis to identify predictor variables (baseline, maternal and prenatal, neonatal and postnatal morbidities) that significantly influenced mortality in preterm infants and to determine the corrected ORs for each of them. In Model 1, only baseline and maternal–prenatal predictors (i.e., variables present before SGA classification or before birth) were included as confounders (Table 1). In Model 2 all variables analyzed in the study (Tables 1, 2) were included as independent or predictor variables, comprising both confounders and potential mediators (i.e., variables occurring after birth that may lie in the causal pathway between SGA status and mortality), with the exception of moderate-severe bronchopulmonary dysplasia and retinopathy of prematurity, as their positivity would be practically indicative of survival. Therefore, moderate-severe bronchopulmonary dysplasia and retinopathy of prematurity analyses were survivor-only analyses. Collinearity was assessed using linear regression. Sensitivity analyses were conducted by restricting the cohort to singleton pregnancies and by redefining SGA using the 3rd percentile rather than the 10th percentile as the cutoff. All statistical analyses were conducted using SPSS software (version 27) and the delta method. A *p*-value <0.05 was considered statistically significant.

**Table 1:** Baseline maternal and neonatal characteristics of preterm infants according to SGA status for gestational age (fenton-2013 curves), stratified into three gestational age groups.

1995–2023 *N* = 11.208 Non- SGA: 9.547 SGA: 1.661 (14.8%)		23 A 27 Weeks			28 A 31 Weeks			32 A 34 Weeks		
		Non-SGA	SGA	*N* = 1.475	Non-SGA	SGA	*N* = 2.844	Non-SGA	SGA	*N* = 6.889
		1,313	162	11.0%	2,507	337	11.8%	5,727	1,162	16.9%
		Median (IQR); %	Median (IQR), %	*p* value; OR (95% CI)	Median (IQR); %	Median (IQR); %	*p* value; OR (95% CI)	Median (IQR); %	Median (IQR); %	*p* value; OR (95% CI)
Baseline										
Period:										
1995–2000	12.2%		11.1%	NS	15.9%	16.0%	NS	16.1%	15.8%	NS
2001–2009	39.0%		33.3%	(0.264)	40.5%	38.6%	(0.777)	38.6%	35.9%	(0.152)
2010–2023	48.8%		55.6%		43.6%	45.4%		45.3%	48.3%	
Center:										
HCP + HCM	53.7%		55.6%	NS	59.3%	56.4%	NS	53.4%	57.0%	**0.023**
HSJD	46.3%		44.4%	(0.663)	40.7%	43.6%	(0.320)	46.6%	43.0%	
	Mis:5		Mis:0		Mis:8	Mis:2		Mis:6	Mis:3	
Maternal prenatal										
Multiple gestation	29.0%		35.8%	NS; 1.36	37.2%	29.5%	**0.005**; 0.70	34.9%	39.3%	**0.005**: 1.21
	Mis:4		Mis:0	(0.97–1.92)	Mis:4	Mis:1	(0.55–0.90)	Mis:19	Mis:2	(1.06–1.37)
Corticoids to mother	84.4%		85.7%	NS; 1.11	83.5%	87.5%	NS; 1.39	66.6%	74.1%	**<0.001** ; 1.44
	Mis:67		Mis:8	(0.69–1.78)	Mis:89	Mis:16	(0.98–1.97)	Mis:296	Mis:58	(1.24–1.66)
Cesarean section^a^	42.6%		64.0%	**<0.001**; 2.39	62.0%	95.2%	**<0.001**; 12.2	52.0%	83.2%	**<0.001**; 4.57
	Mis:17		Mis:1	(1.70–3.36)	Mis:28	Mis:3	(7.34–20.3)	Mis:86	Mis:12	(3.89–5.39)
Preeclampsia^a^	4.3%		22.2%	**<0.001**; 6.30 (3.99–9.93)	11.1%	37.1%	**<0.001**; 4.71 (3.65–6.07)	9.4%	29.3%	**<0.001**; 4.00 (3.43–4.66)
Diabetes in mother	3.0%		3.1%	NS; 1.01 (0.39–2.61)	6.0%	5.9%	NS; 0.98 (0.61–1.59)	8.6%	6.8%	**0.042**; 0.77 (0.61–0.99)
** **Fetal distress^a^ (1)	67 5.1%		20 12.3%	**<0.001**; 2.62 (1.54–4.44)	169 6.7%	49 14.5%	**<0.001**; 2.35 (1.67–3.31)	300 5.2%	108 9.3%	**<0.001**; 1.85 (1.47–2.33)
** **Gestational age	26 (25–27)		26 (25–27)	NS (0.919)	30.14 (29–31)	30.14 (29.3–31)	NS (0.965)	33.86 (33–34.1)	34 (33–34.1)	NS (0.380)
** **Male	54.5%		54.9%	NS; 1.02 (0.73–1.41)	52.1%	56.1%	NS; 1.17 (0.93–1.47)	54.8%	53.7%	NS; 0.958 (0.84–1.09)
NEONATAL										
** **Weight^a^	835 (710–970)		540 (480–600)	**<0.001**	1,375 (1,200–1,600)	850 (740–965)	**<0.001**	2,045 (1,850–2,280)	1,459 (1,280–1,605)	**<0.001**
** **Apgar 1 min ≤5^a^	50.7% Mis:31		59.5% Mis:4	**0.033**; 1.43 (1.02–2.00)	22.1% Mis:23	34.8% Mis:1	**<0.001**; 1.88 (1.47–2.40)	8.9% Mis:51	13.6% Mis:6	**<0.001**; 1.60 (1.32–1.94)
** **Vigorous Reanimation	52.3% Mis:59		59.6% Mis:11	NS; 1.34 (0.95–1.90)	13.7% Mis:95	21.6% Mis:13	<0.001; 1.73 (1.30–2.31)	2.8% Mis:96	4.4% Mis:33	0.004; 1.60 (1.15–2.21)

SGA, small for gestational age; Median, 50th percentile; IQR, interquartile range (25th–75th percentile); OR, odds ratio; 95% CI, 95% confidence interval; NS, not significant; Mis, missings, when present.

Values are presented as median (IQR) or percentage (%).

*p* values in bold indicate statistical significance.

Underlined OR values indicate a consistent increasing or decreasing trend across gestational age groups.

(1) Chi-square test comparing SGA and non-SGA infants across the three gestational age groups: *p* **=** **0.498 (NS).**

a Higher frequency in SGA than in non-SGA infants in all gestational age groups.

**Table 2:** Neonatal morbidity of preterm infants according to SGA status for gestational age (fenton-13 curves), stratified into three gestational age groups.

1995–2023 *N* = 11.208 Non- SGA: 9.547 SGA: 1.661 (14.8%)	23 A 27 Weeks			28 A 31 Weeks			32 A 34 Weeks		
	Non-SGA	SGA	*N* = 1.475	Non-SGA	SGA	*N* = 2.844	Non-SGA	SGA	*N* = 6.889
	1,313	162	11.0%	2,507	337	11.8%	5,727	1,162	16.9%
	Median (IQR); %	Median (IQR); %	*p* value; OR (95% CI)	Median (IQR); %	Median (IQR); %	*p* value; OR (95% CI)	Median (IQR); %	Median (IQR); %	*p* value; OR (95% CI)
Postnatal morbidity									
HMD (2)	781 59.5%	113 69.8%	**0.012**; 1.57 (1.10–2.24)	828 33.0%	94 27.9%	NS; 0.78 (0.61–1.01)	596 10.4%	64 5.5%	**<0.001**; 0.50 (0.38–0.65)
Pneumothorax	9.7%	15.4%	**0.025**; 1.69 (1.06–2.69)	2.6%	2.1%	NS; 0.78 (0.36–1.72)	1.0%	0.8%	NS; 0.80 (0.40–1.63)
IVH (3)	465 35.4%	44 27.2%	**0.037**; 0.68 (0.47–0.98)	267 10.7%	29 8.6%	NS; 0.79 (0.53–1.18)	127 2.2%	57 4.9%	**<0.001**; 2.27 (1.65–3.13)
PVL	2.5%	5.6%	**0.028**; 2.28 (1.07–4.86)	1.2%	0.3%	NS; 0.25 (0.03–1.87)	0.3%	0.2%	NS; 0.66 (0.15–2.87)
PDA (4)	643 49.0%	66 40.7%	**0.048**; 0.72 (0.51–1.00)	413 16.5%	59 17.5%	NS; 1.08 (0.80–1.45)	138 2.4%	35 3.0%	NS; 1.26 (0.86–1.83)
ARF	8.4%	12.3%	NS; 1.54 (0.93–2.56)	1.3%	2.7%	NS; 2.06 (0.98–4.34)	0.6%	0.7%	NS; 1.10 (0.51–2.36)
Sepsis	32.5%	27.8%	NS; 0.80 (0.55–1.15)	10.7%	14.5%	**0.033**; 1.43 (1.03–1.98)	2.2%	2.6%	NS; 1.20 (0.80–1.79)
NEC	8.9%	7.4%	NS; 0.82 (0.44–1.52)	2.8%	5.0%	**0.028**; 1.82 (1.06–3.13)	0.7%	0.9%	NS; 1.43 (0.73–2.81)
GIP	6.7%	5.6%	NS; 0.82 (0.40–0.92)	0.8%	3.0%	**<0.001**; 3.80 (1.76–8.19)	0.2%	0.1%	NS; 0.45 (0.06–3.47)
Death (5)	347 26.4%	88 54.3%	**<0.001**; 3.31 (2.37–4.62)	89 3.6%	30 8.9%	**<0.001**; 2.65 (1.73–4.08)	55 1.0%	13 1.1%	NS; 1.17 (0.63–2.14)
Only alives: 10.586	**966**	**74**	***N*** **=** **1,040**	**2,418**	**307**	***N*** **=** **2,725**	**5,672**	**1,149**	***N*** **=** **6,821**
BPD (36 weeks) (6)	124 12.9%	26 35.1%	**<0.001**; 3.65 (2.19–6.10)	61 2.5%	16 5.2%	**0.008**; 2.12 (1.21–3.72)	13 0.2%	4 0.4%	NS; 1.52 (0.49–4.67)
ROP (7)	472 48.9%	44 59.5%	NS; 1.53 (0.95–2.48)	194 8.0%	78 25.4%	**<0.001**; 3.90 (2.90–5.25)	25 0.1%	5 2.2%	**<0.001**; 25.2 (9.63–66.0)

SGA, small for gestational age; P50, 50th percentile (median); IQR, interquartile range (25th–75th percentile); %, percentage; OR, odds ratio; NS, not significant; Mis, missings, when present.

HMD, hyaline membrane disease; IVH, intraventricular hemorrhage; PVL, periventricular leukomalacia; PDA, patent ductus arteriosus; BPD, moderate-to-severe bronchopulmonary dysplasia; ROP, retinopathy of prematurity.

Values are presented as median (IQR) or percentage (%).

*p* values in bold indicate statistical significance.

Underlined OR values indicate a consistent increasing or decreasing trend across the three gestational age groups.

(2) Chi-square test comparing SGA and non-SGA infants across the three gestational age groups: *p* **=** **0.121 (NS).** (3) Chi-square test comparing SGA and non-SGA infants across the three gestational age groups: *p* **<** **0.001.** (4) Chi-square test comparing SGA and non-SGA infants across the three gestational age groups: *p* **<** **0.001.** (5) Chi-square test comparing SGA and non-SGA infants across the three gestational age groups: *p* **=** **0.456 (NS).** (6) Chi-square test comparing SGA and non-SGA infants across the three gestational age groups: *p* **=** **0.720 (NS).** (7) Chi-square test comparing SGA and non-SGA infants across the three gestational age groups: *p* **<** **0.001.**

## Results

Initially, 12,550 preterm infants between 23 and 34 weeks' gestation were included. Of these, 1,318 were excluded due to some type of malformation, chromosomal abnormality, or metabolic disorder, and 24 were excluded due to missing data essential for their classification. Ultimately, 11,208 preterm infants were analyzed.

Applying the Fenton-13 curves, 1,661 preterm infants were classified as SGA (14.8%) and 9,547 as non-SGA (85.2%). They were divided into three groups according to gestational age, and in none of them were significant differences between the gestational age of the SGA and non-SGA infants, which is essential to consider that, within each group, the SGA and non-SGA infants are comparable.

Tables 1, 2 show that the presence of SGA was associated with cesarean section, preeclampsia, fetal distress, and Apgar score ≤5 at one minute in all the groups analyzed. Corticosteroids administered to the mother increased between 32 and 34 weeks, while maternal diabetes decreased in this group. Vigorous resuscitation was always more necessary in SGA infants but only reached statistical significance from 28 weeks onward. In SGA infants, some conditions may increase or decrease depending on gestational age. Multiple pregnancy decreased between 28 and 31 weeks but increased between 32 and 34 weeks. Hyaline membrane disease increased between 23 and 27 weeks but decreased between 32 and 34 weeks, the opposite of intraventricular hemorrhage. Pneumothorax and periventricular leukomalacia only increased between 23 and 27 weeks, while patent ductus arteriosus decreased in this group. Necrotizing enterocolitis, sepsis, and gastrointestinal perforation increased between 28 and 31 weeks. Acute renal failure showed no relationship with SGA in any group. In SGA survivors, moderate-severe bronchopulmonary dysplasia and retinopathy of prematurity were more common in all groups, but moderate-severe bronchopulmonary dysplasia only reached statistical significance between 27 and 31 weeks, and retinopathy of prematurity only after 28 weeks Missing data for each variable, when present, were reported in Tables 1, 2. The proportion of missing data was negligible.

**Figure 1:**
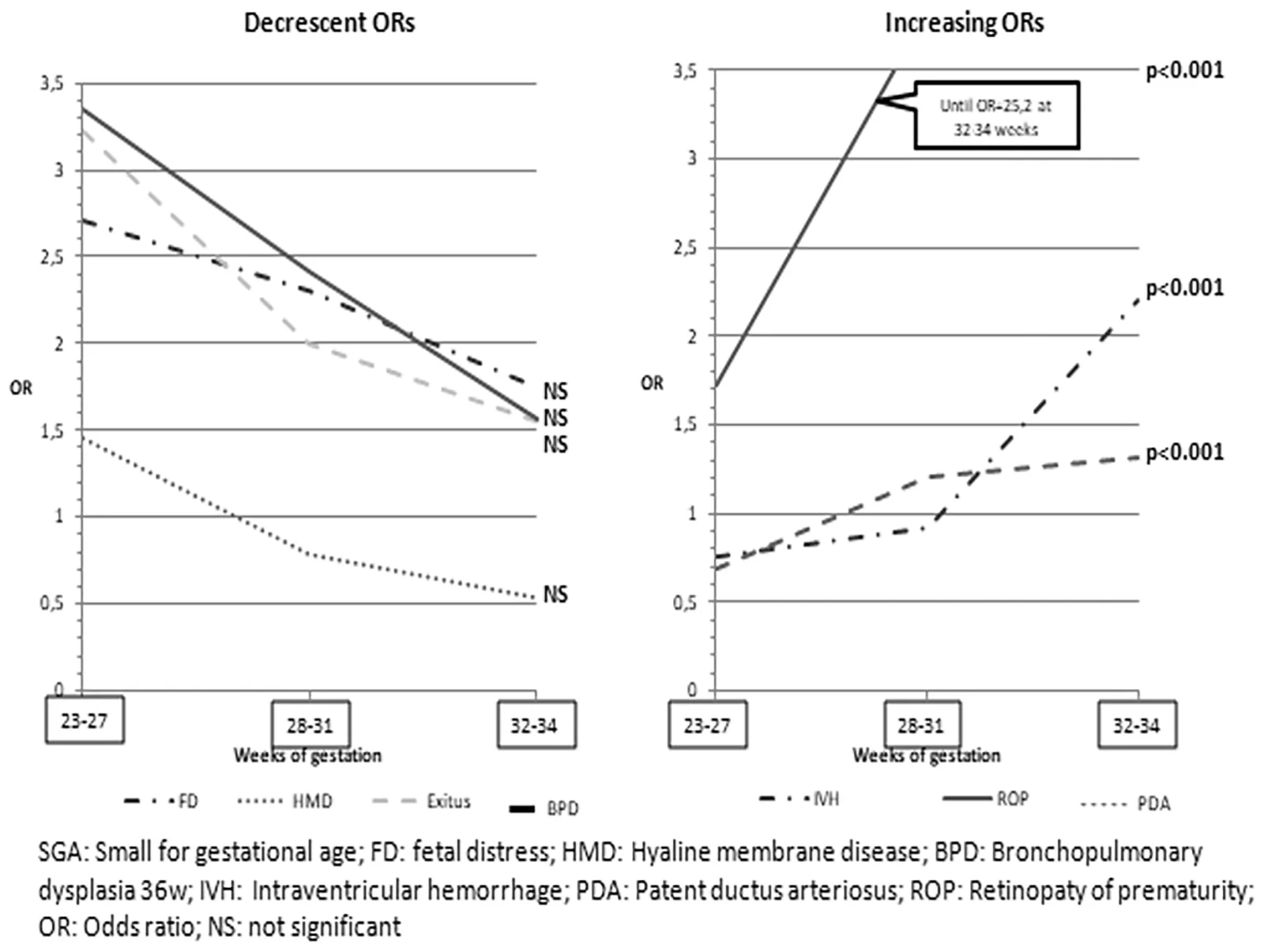
Trends in various morbidities between SGA and non-SGA infants assessed using odds ratios (ORs) according to fenton-2013 curves.

Some variables showed a sustained trend across the three gestational age groups, with their odds ratios (ORs) remaining consistent in SGA compared to non-SGA. As gestational age increased, the odds ratios (ORs) for maternal preeclampsia (6.30 to 4.00), maternal diabetes (1.01 to 0.77), fetal distress (2.62 to 1.86), hyaline membrane disease (1.57 to 0.50), death (3.31 to 1.17), and moderate-severe bronchopulmonary dysplasia (3.65 to 1.52) decreased. Conversely, the ORs for maternal corticosteroid administration (1.11 to 1.44), intraventricular hemorrhage (0.68 to 2.27), patent ductus arteriosus (0.72 to 1.26), and retinopathy of prematurity (1.54 to 25.21) increased. However, these trends in neonatal clinical conditions were only significant for intraventricular hemorrhage, patent ductus arteriosus, and retinopathy of prematurity (Figure 1).

According to interaction testing, gestational age appeared to modify the associations between birth weight status and hyaline membrane disease, intraventricular hemorrhage, and mortality (Table 3). This table also presents the sensitivity analysis in which SGA was redefined using the 3rd percentile rather than the 10th percentile as the cutoff. The overall pattern of associations remained consistent (Table 3), except for mortality (*p* = 0.072) and gastrointestinal perforation (*p* = 0.551), likely reflecting the smaller number of infants classified as SGA. This lack of consistency was further confirmed by the additive interaction measures (Table 4) only for gastrointestinal perforation. Therefore, the outcomes showing the strongest evidence of interaction were hyaline membrane disease, intraventricular hemorrhage, and death.

**Table 3:** Logistic regression models included an interaction term between birth weight status (SGA vs. non-SGA) and gestational age group (three categories). Interaction *p*-values were calculated for all outcomes after adjustment for potential confounders.

1995–2023	Percentile 10 SGA: 1,661 (14.8%)			Percentile 3 SGA: 513 (4.6%)		
*N* = 11,208						
	Birth weight status	Gestational age group	Interaction	Birth weight status	Gestational age group	Interaction
Morbidity						
Hyaline membrane disease	**<0**.**001**	**<0**.**001**	**<0**.**001**	**<0**.**001**	**<0**.**001**	**0**.**023**
Pneumothorax	0.597	**<0**.**001**	0.278	0.504	**<0**.**001**	0.531
Intraventricular hemorrhage	**<0**.**001**	**<0**.**001**	**<0**.**001**	**<0**.**001**	**<0**.**001**	**<0**.**001**
Periventricular leukomalacia	0.802	**<0**.**001**	0.367	0.847	**<0**.**001**	0.988
Patent ductus arteriosus	0.826	**<0**.**001**	0.109	0.925	**<0**.**001**	0.535
Acute renal failure	0.742	**<0**.**001**	0.707	**0**.**002**	**<0**.**001**	0.485
Sepsis culture-proven	0.202	**<0**.**001**	0.093	**0**.**012**	**<0**.**001**	0.064
Necrotizing enterocolitis	0.789	**<0**.**001**	0.394	0.177	**<0**.**001**	0.882
Gastrointestinal perforation	0.481	**<0**.**001**	**0**.**023**	0.655	**<0**.**001**	0.551
Death	0.269	**<0**.**001**	**0**.**024**	0.131	**<0**.**001**	0.072

Potential confounders: period of birth, center, multiple pregnancy, prenatal corticosteroids, preeclampsia, maternal diabetes, fetal distress, cesarean section, sex, Apgar score at one minute ≤5, need for vigorous resuscitation (with endotracheal intubation).

Birth weight status was defined using either the 10th or the 3rd percentile as the cutoff.

*p* values in bold indicate statistical significance.

**Table 4:** Effect modification analysis between birth weight status (SGA and non-SGA) and gestational age group (3 groups), including calculation of both additive interaction measures (OR10, OR01, OR11, RERI, AP, S) with adequate adjustment for confounders.

Outcome	OR10	OR01	OR11	RERI	AP	S
	aOR (95% CI)	aOR (95% CI)	aOR (95% CI)	aOR (95% CI)	aOR (95% CI)	aOR (95% CI)
HMD						
Study 1	0.56 (0.42–0.74)	2.15 (1.81–2.55)	2.61 (1.74–3.90)	0.90 (−0.15–1.96)	0.35 (0.06–0.63)	2.28 (0.46–4.10)
Study2	0.33 (0.24–0.44)	6.71 (5.59–8.06)	8.58 (5.69–12.93)	2.54 (−0.92–5.99)	0.30 (0.00–0.59)	1.50 (0.79–2.22)
IVH						
Study 1	0.86 (0.56–1.34)	3.31 (2.70–4.06)	2.11 (1.39–3.21)	−1.06 (−2.08- −0.04)	−0.50 (−1.13–0.13)	0.51 (0.12–0.98)
Study 2	2.16 (1.51–3.10)	13.76 (10.57–17.91)	7.76 (4.89–12.31)	−7.16 (−11.08- −3.23)	−0.92 (−1.71- −0.13)	0.49 (0.26–0.72)
GIP						
Study 1	3.73 (1.63–8.55)	8.56 (4.98–14.70)	8.02 (3.43–18.73)	−3.27 (−9.89–3.34)	−0.408 (−1.45–0.64)	0.68 (0.11–1.26)
Study 2	0.46 (0.06–3.62)	32.98 (16.19–67.14)	29.22 (3.31–258.22)	−3.22 (−25.40–18.97)	−0.110 (−0.94–0.72)	0.90 (0.21–1.59)
Death						
Study 1	3.10 (1.89–5.09)	7.19 (5.34–9.68)	29.67 (19.01–46.31)	20.37 (8.38–32.35)	0.69 (0.56–0.81)	3.46 (1.99–4.92)
Study 2	1.44 (0.74–2.79)	20.51 (13.79–30.50)	86.40 (51.23–145.73)	65.45 (25.19–105.71)	0.76 (0.66–0.87)	4.28 (2.50–6.06)

HMD, hyaline membrane disease; IVH, intraventricular hemorrhage; GIP, gastrointestinal perforation.

Potential confounders: period of birth, center, multiple pregnancy, prenatal corticosteroids, preeclampsia, maternal diabetes, fetal distress, cesarean section, sex, Apgar score at one minute ≤5, need for vigorous resuscitation (with endotracheal intubation); OR: adjusted odds ratio; OR10: aOR SGA status; OR01: aOR gestational age study; OR11: aOR interaction; RERI: Relative Excess Risk due to Interaction; AP: Attributable Proportion due to interaction; S: Synergy Index.

Study 1: comparison between 23 and 27 gestational age group (value 1) and 28–31 gestational age group (value 0).

Study 2: comparison between 23 and 27 gestational age group (value 1) and 32–34 gestational age group (value 0).

Interpretation of Additive Interaction Measures.

- RERI estimates the excess risk (aOR) attributable exclusively to the interaction between the two exposures. RERI = 0: no additive interaction. RERI > 0: positive interaction (synergism). RERI < 0: negative interaction (antagonism).

- AP represents the proportion of the excess risk (aOR) among subjects exposed to both factors that is attributable to their interaction. For example, AP = 0.70 indicates that 70% of the excess risk (aOR) observed among individuals exposed to both factors may reflect their interaction rather than from their independent effects.

- S compares the observed combined effect with the expected effect under an additive model. S = 1: no interaction. S > 1: positive interaction (synergism). S < 1: negative interaction (antagonism).

Results

1. Hyaline Membrane Disease (HMD).

Study 1. Low gestational age (value 1) increased the risk of HMD (OR01 = 2.15), whereas SGA alone was not associated with an increased risk. However, when both factors were present simultaneously, the observed risk exceeded that expected under an additive model. All three additive interaction measures (RERI > 0, AP > 0, and S > 1) indicated a moderate positive interaction, suggesting that 35% of the excess risk was attributable to the interaction between the two factors.

Study 2. This analysis showed the previous pattern, but 30% of the excess risk was attributable to the interaction.

2. Intraventricular Hemorrhage (IVH).

Study 1. Although low gestational age increased the risk of IVH, the combined effect of both exposures was lower than expected under an additive model. All three interaction measures consistently indicated negative interaction (antagonism).

Study 2. The antagonistic interaction was even more pronounced. Each factor independently increased the risk of IVH; however, when both exposures were present simultaneously, the combined effect was substantially lower than expected. Again, all three additive interaction measures consistently demonstrated negative interaction (antagonism).

3. Gastrointestinal perforation (GIP).

Study 1. Although both SGA status and gestational age were independently associated with gastrointestinal perforation, the additive interaction measures did not support effect modification. The point estimates suggested a weak negative interaction (RERI < 0, AP < 0, and S < 1), but all 95% confidence intervals included the null values, indicating no statistically significant additive interaction.

Study 2. Similar findings were observed in the second analysis. Overall, gestational age does not appear to meaningfully modify the association between SGA and gastrointestinal perforation on the additive scale.

4. Mortality (Death).

Study 1. A strong positive interaction was observed. The combined effect of both factors was considerably greater than expected under an additive model. 69% of the excess mortality was attributable to the interaction.

Study 2. This was the most striking finding of the study. The combined effect (OR11 = 86.40) was dramatically greater than expected. All three additive interaction measures demonstrated very strong synergism, with a markedly elevated RERI=65.45, AP = 76%, and S = 4.28. From a clinical perspective, the coexistence of both factors identifies a subgroup of newborn infants with an exceptionally high risk of death, substantially exceeding the risk expected from the sum of their independent effects.

Fewer statistically significant associations were observed, particularly for mortality, likely reflecting the smaller number of infants classified as SGA.

The findings of the additive interaction measures (Table 4) suggest that gestational age may modify the association between SGA and these outcomes. Hyaline membrane disease demonstrated moderate synergism, suggesting that the coexistence of SGA and low gestational age groups increases the risk more than expected from their independent effects. Intraventricular hemorrhage consistently demonstrated negative interaction (antagonism), indicating that the combined effect of both exposures was smaller than expected under an additive model. Mortality showed marked synergism, particularly in the analysis comparing low gestational age (23–27 weeks) with high gestational ages (32 to 34 weeks), in which 69% to 76% of the excess mortality was attributable to the interaction between the two exposures. Gastrointestinal perforation consistently showed no evidence of additive interaction between SGA status and gestational age.

622 preterm infants (5.5%) died, 131 (7.9%) of the SGA group and 491 (5.1%) of the other non-SGA groups, with *p* = 0.001 and OR:1.58 (1.29–1.93. Logistic regression analysis of baseline and maternal–prenatal factors associated with mortality identified protective factors that remained significant in the extended model (birth period, antenatal corticosteroids, and gestational age). In contrast, factors initially associated with an increased risk of mortality (cesarean section, fetal distress, and male sex) were excluded from the model after adding neonatal factors and postnatal morbidities (Table 5). The extended multivariate logistic regression analysis included 91.6% of the sample and reflected 51.1% of the variance (Table 6). The variables with an independently significant influence on the prediction of mortality in premature infants from 23 to 34 weeks were: Apgar 1 min ≤5 (OR:1.92), need for vigorous resuscitation (OR:1.82), presence of SGA (OR:3.13), hyaline membrane disease (OR:1.43), pneumothorax (OR:4.26), intraventricular hemorrhage (OR:2.87), acute renal failure (OR:6.21), necrotizing enterocolitis (OR:3.27) and gastrointestinal perforation (OR:2.20). Protective factors included: a more recent period of birth (OR:0.78), higher gestational age (OR:0.65) and prenatal corticosteroid administration to the mother (OR:0.45). The effect of patent ductus arteriosus (OR:0.45) is noteworthy, indicating that its diagnosis is associated with lower mortality. Variables not included in the logistic regression model because they were not statistically significant were center, multiple pregnancy, cesarean section, preeclampsia, maternal diabetes, fetal distress, sex, birth weight, periventricular leukomalacia, and sepsis.

**Table 5:** Logistic regression analysis looking for baseline and maternal - prenatal predictors factors of mortality, in all the sample (singles + multiples) and only in singles, for performing a sensitivity analysis. Collinearity is shown (non-collinearity: tolerance should be >0.1).

1995–2023	Singleton + multiple pregnancies				Only singleton pregnancies^a^			
	Incluidos: 94.3% R^2^ Nagelkerke = 0,393				Incluidos: 94.4% R2 = 0,346			
	p	OR	95%CI	Tolerance	p	OR	95%CI	Tolerance
Baseline								
Period: 1995–2000 (0) 2001–2009 (1) 2010–2023 (2)	<0.001	0.73	0.64–0.84	0.960	0.002	0.79	0.67–0.92	0.957
Center: HCP + HCM (0) HSJD (1)	NS			0.942	NS			0.939
Maternal - prenatal								
Multiple gestation	NS			0.922	NS			–
Corticoids to mother	<0.001	0.49	0.39–0.62	0.905	<0.001	0.46	0.35–0.60	0.904
Cesarean section	0.030	1.25	1.02–1,53	0.895	0.001	1.48	1.16–1.89	0.884
Preeclampsia	NS			0.892	NS			0.865
Diabetes in mother	NS			0.978	NS			0.980
Fetal distress	<0.001	2.68	1.97–3.66	0.957	<0.001	2.34	1.65–3.30	0.947
Gestational age	<0.001	0.56	0.54–0.58	0.947	<0.001	0.59	0.57–0.62	0.946
Male	0.036	1.23	1.01–1.49	0.997	NS			0.995

NS, not significant.

(): subgroup values; if not specified: 0 = no, 1 = yes.

- Variables initially considered: all the variables included in the analysis (column 1).

- Variables in the final model: significant variables, with OR and 95%CI.

- Variables excluded: not significant variables (*p*>=0.05).

a Sensitivity analysis.

**Table 6:** Logistic regression analysis looking for predictors factors of mortality, in all the sample (singles + multiples) and only in singles, for performing a sensitivity analysis. Collinearity is shown (non-collinearity: tolerance should be >0.1).

1995–2023	Singleton + multiple pregnancies				Only singleton pregnancies^a^			
	Included: 91.6% R^2^ Nagelkerke = 0,511				Included: 91.5% R^2^ Nagelkerke = 0,472			
	p	OR	95% CI	Tolerance	p	OR	95% CI	Tolerance
Baseline								
Period: 1995–2000 (0) 2001–2009 (1) 2010–2023 (2)	0.001	0.78	0.62–0,91	0.931	0.027	0.81	0.68–0.98	0.929
Center: HCP + HCM (0) HSJD (1)	NS			0.929	NS			0.924
Maternal - prenatal								
Multiple gestation	NS			0.891	NS			–
Corticoids to mother	<0.001	0.45	0.34–0.59	0.878	<0.001	0.40	0.29–0.55	0.869
Cesarean section	NS			0.840	NS			0.811
Preeclampsia	NS			0.844	NS			0.792
Diabetes in mother	NS			0.963	NS			0.954
Fetal distress	NS			0.926	NS			0.915
Gestational age	<0.001	0.65	0.62–0.68	0.194	<0.001	0.69	0.65–0.73	0.212
Male	NS			0.971	NS			0.975
Neonatal								
Weight	NS			0.208	NS			0.203
Small for gestational age	<0.001	3.13	2.36–4.17	0.600	<0.001	2.83	2.0–4.0	0.586
Apgar 1 min ≤5	<0.001	1.92	1.50–2.47	0.688	<0.001	2.51	1.86–3.39	0.683
Vigorous Reanimation	<0.001	1.82	1.38–2.40	0.594	<0.001	1.84	1.33–2.56	0.598
Postnatal morbidity								
Hyaline membrane disease	<0.001	1.43	1.11–1.95	0.694	(0.089)	1.31	0.96–1.77	0.690
Pneumothorax	<0.001	4.26	2.93–6.21	0.931	<0.001	3.10	1.96–4.89	0.931
Intraventricular hemorrhage	<0.001	2.87	2.23–3.70	0.809	<0.001	2.66	1.96–3.61	0.799
Periventricular leukomalacia	NS			0.983	NS			0.979
Patent ductus arteriosus	<0.001	0.45	0.34–0.59	0.719	<0.001	0.47	0.34–0.66	0.718
Acute renal failure	<0.001	6.21	4.16–9.27	0.911	<0.001	7.08	4.37–11.5	0.914
Sepsis culture-proven	NS			0.819	NS			0.828
Necrotizing enterocolitis	<0.001	3.27	2.14–4.99	0.843	<0.001	3.50	2.12–5.76	0.836
Gastrointestinal perforation	0.003	2.20	1.31–3.69	0.838	0.035	1.99	1.05–3.77	0.839

NS, not significant.

(): subgroup values; if not specified: 0 = no, 1 = yes.

- Variables initially considered: all the variables included in the analysis (column 1).

- Variables in the final model: significant variables, with OR and 95%CI.

- Variables excluded: not significant variables (*p* ≥ 0.05).

a Sensitivity analysis.

The cohort includes both singleton and multiple pregnancies. A sensitivity analysis of the logistic regression restricted to singleton pregnancies showed that the results were very similar to those obtained in the entire cohort (Table 6). Therefore, the main findings can be considered robust.

## Discussion

The main problem encountered when classifying a newborn as SGA is deciding which growth charts to use (fetal or neonatal charts) and choosing the cutoff point for classifying small for gestational age (10th percentile, 3rd percentile, -2 standard deviations) based on gestational age and sex (13). Deorari et al. described the overdiagnosis of SGA according to the curves used (14). The Fenton-2013 (3) charts used in the present study are growth references derived from large population-based datasets, whereas the INTERGROWTH-21st (4) standards and the recently published third-generation Fenton-2025 (5) charts were developed to represent optimal fetal and preterm growth under selected conditions. Consequently, the choice of chart may influence the number of infants classified as SGA and, therefore, the estimated associations with neonatal outcomes. In a recent publication (15) among preterm infants born at 24 to 31 weeks of gestation, the Fenton-2013 growth charts showed the best discrimination of mortality between SGA and non-SGA in our country. Therefore, the Fenton-13 neonatal growth charts were chosen to classify SGA status in this study.

No differences were found in relation to sex. The results of the present study support the idea that in SGA placental insufficiency remains a plausible hypothesis rather than a demonstrated mechanism. Placental insufficiency is sometimes conditioned by maternal preeclampsia, which worsens fetal well-being (greater neonatal depression with lower Apgar scores at one minute) and leads to more cesarean sections (16). However, the present study was not designed to investigate the underlying mechanisms responsible for SGA. No placental histopathology, fetal Doppler evaluation, or biomarkers of placental dysfunction were available. Placental insufficiency cannot be confirmed in the present cohort. SGA is a heterogeneous clinical condition that includes infants with different etiologies, including placental dysfunction, maternal disorders, genetic influences, constitutional small size, and other fetal conditions. The observed gestational age-dependent differences should be interpreted as clinical associations rather than evidence of a specific underlying biological mechanism. Future studies integrating placental pathology and functional assessment are warranted to clarify these mechanisms.

The study included infants born over a 29-year period, during which substantial advances occurred in neonatal intensive care. Although birth period was included as an adjustment variable in all multivariable models to account for these secular trends, residual confounding related to changes in clinical practice cannot be completely excluded. In addition, all multivariable models were adjusted for center, thereby accounting for potential differences in clinical practice among participating hospitals.

Consistent with the descriptions in the literature, mortality was always increased in the extremely preterm and very preterm infants with SGA in our study (10, 12, 17–19). Boghossian et al. (12) studied the influence of SGA in preterm infants aged 22–29 weeks, using data from the Vermont Oxford Network, finding an increase in mortality and morbidity in SGA. In the literature, moderate-severe bronchopulmonary dysplasia (12, 18, 20) and retinopathy of prematurity (12, 19) increase in SGA. In our series, both also increased, but moderate-severe bronchopulmonary dysplasia is only significantly higher between 23 and 31 weeks, while retinopathy of prematurity is higher from 28 weeks onwards.

The elevation of hyaline membrane disease (12, 17) described in the literature, in our series was only significant between 23 and 27 weeks, while it decreased to become significantly lower at 32–34 weeks. This could be due to the condition causing SGA, which may increase fetal stress and endogenous cortisol production, or to the greater administration of antenatal corticosteroids to the mother (78.0% in SGA vs. 73.5% in non-SGA; *p* < 0.001).

Boghossian et al. (12) stated that patent ductus arteriosus decreased in early gestational ages and then increased. In our series, the same percentage relationship was found, but the decrease only reached statistical significance between 23 and 27 weeks. The literature reports both an increase in intraventricular hemorrhage (10) and an absence of relationship (11) and it has even been suggested that it could be decreased (12). In our study, a percentage decrease in intraventricular hemorrhage was found between 23 and 27 weeks, similar to Boghossian's work (12), while it only increased significantly between 32 and 34 weeks. These results are contrary to those of hyaline membrane disease. Being SGA does not influence periventricular leukomalacia except for the slight increase between 23 and 27 weeks. SGA has been described as being associated with an increase in late-onset sepsis (8, 12), which also occurred in our series between 28 and 31 weeks.

In the literature, SGA preterm infants present with more conditions related to the hypoxic-ischemic event, such as acute renal failure, gastrointestinal perforation, and necrotizing enterocolitis (12, 17, 21). In our study, both necrotizing enterocolitis and gastrointestinal perforation only occurred between 28 and 31 weeks of gestation. D'Inca et al. (22) already described, in a porcine model of term and preterm infants, that intrauterine growth restriction was associated with a longer and thinner intestine at birth, especially the ileum, requiring more time for intestinal adaptation and bacterial colonization. In our study, acute renal failure was always proportionally higher in SGA infants, but this difference never reached statistical significance nor did show any trend.

Analyses of the sustained trends in odds ratios (ORs) for SGA infants compared to non-SGA infants across the three gestational age groups revealed that, if the ORs were increasing, SGA preterm infants had a higher probability of presenting these morbidities as gestational age increased, contrary to what would occur if the ORs were decreasing. This was particularly true for intraventricular hemorrhage, patent ductus arteriosus, and retinopathy of prematurity, all of which showed statistically significant increasing trends (*p* < 0.001). Gestational age appeared to modify the associations between SGA status and selected neonatal outcomes, including hyaline membrane disease, intraventricular hemorrhage, and mortality.

To determine the true influence of SGA on mortality in preterm infants aged 23–34 weeks, it is essential to consider all factors possibly related to their hospital mortality. The results obtained in the multivariate analysis confirm that SGA status according to Fenton-13 increased the probability of death in these preterm infants by 3.13 times. Guellec et al. (23) reported an increase of 2.99 times. A more recent birth period is associated with a lower risk of mortality, whereas the center is not significantly associated with mortality. The protective effect of prenatal corticosteroids should be noted. The seemingly paradoxical protective effect of patent ductus arteriosus could be linked to the longer life expectancy at diagnosis, which already implies lower mortality. The additive interaction measures showed that the coexistence of SGA and low gestational age group identifies an exceptionally vulnerable subgroup of preterm infants. Acknowledging that the mortality models include several postnatal morbidities that may lie on the causal pathway between SGA and death, these variables were included for prognostic rather than causal inference purposes.

One limitation of the study is to be retrospective, although the data were collected prospectively. It is worth noting the large sample analyzed in three hospitals that followed the same obstetric and neonatal protocols throughout the 29 years of follow-up. However, a drawback could be the need to maintain detailed data collection over a very long period with changes in care. This is supported by the observed reduction in mortality in more recent years, while mortality rates remained similar across centers. Nevertheless, disparities in data collection existed in both the SGA and non-SGA groups, and the comparative study remains valid, even though the absolute incidence figures are lower than the actual figures. The magnitude of the observed associations might differ if more severe definitions of growth restriction were applied (below Percentile 3th). Another limitation of the study is that the analyses of bronchopulmonary dysplasia and retinopathy of prematurity were restricted to survivors.

## Conclusions

The magnitude of the adverse prognosis associated with SGA is not constant throughout prematurity. Gestational age modifies several important associations. These findings may contribute to more individualized risk assessment and support future studies exploring gestational age-specific management strategies.

## Data Availability

The original contributions presented in the study are included in the article/Supplementary Material, further inquiries can be directed to the corresponding author.
